# A standardized nomenclature for the rods and cones of the vertebrate retina

**DOI:** 10.1371/journal.pbio.3003157

**Published:** 2025-05-07

**Authors:** Tom Baden, Juan M. Angueyra, Jenny M. Bosten, Shaun P. Collin, Bevil R. Conway, Fabio Cortesi, Karin Dedek, Thomas Euler, Iñigo Novales Flamarique, Anna Franklin, Silke Haverkamp, Almut Kelber, Stephan C.F. Neuhauss, Wei Li, Robert J. Lucas, Daniel C. Osorio, Karthik Shekhar, Dario Tommasini, Takeshi Yoshimatsu, Joseph C. Corbo

**Affiliations:** 1 Sussex Neuroscience, University of Sussex, Brighton, United Kingdom; 2 Department of Biology and Brain and Behavior Institute, University of Maryland, College Park, Maryland, United States of America; 3 School of Agriculture, Biomedicine and Environment, La Trobe University, Bundoora, Australia,; 4 Laboratory of Sensorimotor Research, National Eye Institute, Bethesda, Maryland, United States of America; 5 Faculty of Science, School of the Environment, University of Queensland, St Lucia, Australia,; 6 Neurosensory/Animal Navigation, Carl von Ossietzky Universität Oldenburg, Oldenburg, Germany; 7 Centre for Ophthalmology, University of Tübingen, Tübingen, Germany; 8 Department of Biological Sciences, Simon Fraser University, Burnaby, Canada; 9 Department of Computational Neuroethology, Max Planck Institute for Neurobiology of Behavior - Caesar, Bonn, Germany; 10 Department of Biology, Lund University, Lund, Sweden; 11 Department of Molecular Life Sciences, University of Zurich, Zurich, Switzerland; 12 National Eye Institute, Bethesda, Maryland, United States of America; 13 Centre for Biological Timing and Division of Neuroscience, School of Biological Sciences, University of Manchester, Manchester, United Kingdom; 14 Department of Chemical and Biomolecular Engineering, University of California Berkeley, Berkeley, California, United States of America; 15 Department of Ophthalmology and Visual Sciences, Washington University School of Medicine, St. Louis, Missouri, United States of America; 16 Department of Pathology and Immunology, Washington University School of Medicine, St. Louis, Missouri, United States of America

## Abstract

Vertebrate photoreceptors have been studied for well over a century, but a fixed nomenclature for referring to orthologous cell types across diverse species has been lacking. Instead, photoreceptors have been variably—and often confusingly—named according to morphology, presence/absence of ‘rhodopsin’, spectral sensitivity, chromophore usage, and/or the gene family of the opsin(s) they express. Here, we propose a unified nomenclature for vertebrate rods and cones that aligns with the naming systems of other retinal cell classes and that is based on the photoreceptor type’s putative evolutionary history. This classification is informed by the functional, anatomical, developmental, and molecular identities of the neuron as a whole, including the expression of deeply conserved transcription factors required for development. The proposed names will be applicable across all vertebrates and indicative of the widest possible range of properties, including their postsynaptic wiring, and hence will allude to their common and species-specific roles in vision. Furthermore, the naming system is open-ended to accommodate the future discovery of as-yet unknown photoreceptor types.

## Abstract 2

Vertebrate photoreceptors have been studied for well over a century, but a fixed nomenclature for referring to orthologous cell types across species has been lacking. In this Consensus View, the authors propose a new consolidated and pan-vertebrate naming system for the rod and cone photoreceptors of the eye.

## Introduction

Vertebrate vision begins with the rod and cone photoreceptors that line the back of the eye. Rods are generally associated with low-light vision while cones gradually take over as light levels increase. The common ancestor of extant vertebrates likely possessed a photoreceptor system consisting of one rod and four cone types, based on the shared presence of these five cell types in present-day jawed and jawless vertebrates [1,2]. However, this is not reflected in the current nomenclature; rather, current naming systems for vertebrate ciliary photoreceptors represent a grab bag of species-specific schemes, typically based on a single functional, morphological, or molecular feature. For example, the cone types of the human eye are often referred to as red/L, green/M, and blue/S. This naming system references the wavelength of the cones’ maximal spectral sensitivity as imparted by the visual pigment (opsin + 11-*cis* retinal chromophore) [3,4]. Since the 1990’s [5–7], vertebrate visual photoreceptors have commonly been named according to the opsin that they express: RH1 (rods) and LWS, RH2, SWS2, SWS1 (cones). These ancient gene sub-families were already present in the last common vertebrate ancestor [1], and exhibit remarkable evolutionary conservation, both in the spectral ranges of the visual pigments, and, as we explain here, in the cell types in which they are expressed. Even so, naming photoreceptor types according to the opsin they express can be problematic. For example, human red and green cones differ by a single feature, namely which of two paralogues of the LWS opsin gene is expressed [8]. Meanwhile, human L and M cones derive from a single ancestral photoreceptor type, and homologs of this cell type are present in the eyes of most extant vertebrates [9]. Why, then, should photoreceptors derived from this ancestral type not share a common designation across species?

**Fig 1:**
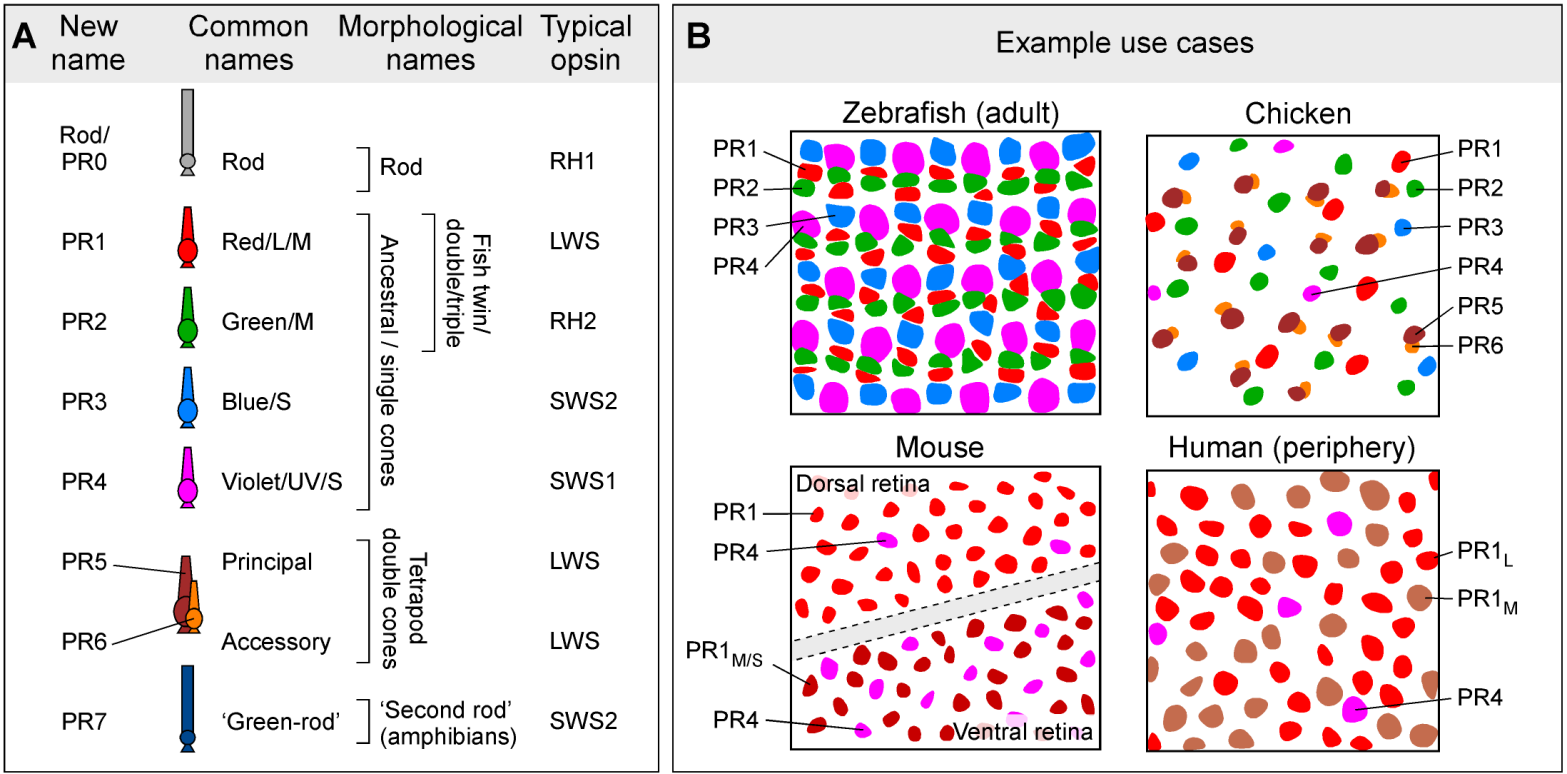
Summary of the proposed new naming system for vertebrate photoreceptors. **(A)** Summary of proposed naming system for vertebrate photoreceptors (left) and its relationship to prior systems [4,9,19,20]. The proposed new system (e.g., PR1, or ‘type 1 photoreceptor’ instead of ‘red’, ‘L/M’, LWS cone, etc.) is intended to capture the conserved ancestral identity of each photoreceptor type. By contrast, various existing naming conventions reference selected spectral, morphological or molecular traits that are not consistent across species (discussed, e.g., in [9]). **(B)** Examples of how the new photoreceptor names are spatially organized in four well-studied vertebrate species. Rods (PR0) are omitted for simplicity. Coloring based on ancestral photoreceptor identity (compare **A** and **B**). PR1_M/S_ refers to PR1 cones that co-express LWS and SWS1 opsins in the ventral mouse retina [21], while human PR1_L_ and PR1_M_ denote the spectral sensitivity of the LWS opsin paralog expressed.

There are numerous other examples of inconsistencies in vertebrate photoreceptor naming, which can cause confusion (discussed in detail below). The field would therefore benefit from greater clarity and consistency in photoreceptor nomenclature. Herein, we propose an updated, unified naming system that defines vertebrate rods and cones based on their evolutionary history, informed by the functional, anatomical, developmental, and molecular identities of the cell types. Following prior studies of other retinal cell classes, we propose a simple naming system in which the photoreceptor types are designated with two letters (‘PR’ for ‘photoreceptor’) followed by a number (see Fig 1). Optionally, depending on context, we propose that additional information (e.g., opsin type, wavelength of maximal sensitivity, chromophore usage etc.) can be appended as a subscript: PR1_L_; PR1_LWS_; PR1_LWS-560 nm_; PR1_LWS-A2_ etc.). Thus, cones derived from the four ancestral single cone types will be designated PR1–PR4 and rods will be PR0 (Fig 1A). Prior studies have shown that retinal cell types including photoreceptors typically occur in regular spatial mosaics, in which somas of a given photoreceptor type are non-randomly distributed with minimal spacing between neurons of the same type [10,11]. Accordingly, we propose mosaic spacing as a working definition of photoreceptor type, similar to the approach used for defining other retinal neuron types (e.g., Refs [12–14]). Under this definition, regional variation in photoreceptor morphology or gene expression can occur in a single photoreceptor type, without needing to designate regional variants (e.g., foveal versus peripheral cones in human) as distinct cell types. Our classification also includes the tetrapod double cone, which consists of principal (PR5) and accessory (PR6) members, and the so-called ‘green rod’ of amphibians (PR7). The two members of the double cone likely arose in the common ancestor of tetrapods, whereas the ‘green rod’ appears to have arisen early in the amphibian lineage [15]. According to this system, zebrafish retinas possess PR0–PR4 [16], while chicken retinas have PR0–PR6 [17]. Eutherian mammals, which include mice and humans, have PR0, PR1, and PR4 types [8,18]. Depending on context, special notation may be useful to distinguish unique subtypes of photoreceptors, such as human ‘green/M’ and ‘red/L’ cones, which differ only in the expression of paralogous LWS opsin genes and can therefore be designated PR1_L_ and PR1_M_ (or PR1_LWS-560 nm_ and PR1_LWS-530 nm_), respectively. We acknowledge that such a naming system may prove cumbersome in certain contexts, and we accordingly suggest that authors present the ‘full’ name in the introduction of a manuscript, thereafter reverting to a shorthand designation for individual types as needed. The naming system we propose here is meant to be open-ended with additional photoreceptor types being added in the future as they are discovered.

Below, we outline in more detail the need for an updated photoreceptor nomenclature, how our new proposed system applies across species, and how we hope these new names can be embedded into the literature to provide clarity in the field.

## The problem with the current vertebrate photoreceptor nomenclature

In general, the currently used names of photoreceptor types fall into two major groups: those that refer to spectral properties and/or the expressed type(s) of opsin, and those that describe morphological properties, such as the shape of their outer segment, their size, or whether they occur singly or as a pair of cells. Several issues arise from naming photoreceptors this way.

### Opsins and their spectral properties are poor indicators of cone identity

Naming photoreceptors according to their spectral properties (e.g., ‘red’) or the opsin they express (e.g., LWS) is problematic because such features are subject to routine variation within and between species, as exemplified by variation in peak spectral sensitivity within opsin classes [4]. Comparison of humans, zebrafish, and mice illustrates the central problem. Humans and zebrafish have ‘red’, ‘green’, and ‘blue’ cones, while zebrafish additionally have ultraviolet (UV) cones [9]. Yet, human ‘green’ and ‘blue’ cones are evolutionarily unrelated to zebrafish ‘green’ and ‘blue’ cones [22]. The opsins of human ‘green’ and ‘red’ cones are orthologous to zebrafish ‘red’ (all express LWS), and human ‘blue’ is orthologous to zebrafish UV (both express short-wavelength sensitive SWS1 opsin). Another example of the deficiencies of a spectrally based naming system is provided by mouse ‘green’ cones. In mice, green cones exclusively express their ‘green-shifted’ LWS opsin in the dorsal retina (i.e., PR1_LWS_) whereas they co-express LWS and SWS1 opsin in the ventral retina (PR1_LWS/SWS1_) [21,23,24]. The same ancestral neuron type PR1 therefore transitions from ‘functionally green’ to ‘functionally UV’ along the dorsal-ventral axis of the retina. Moreover, mice retain the ancestral UV-sensitive cone type PR4 (PR4_SWS1_), which like the SWS1-coexpressing PR1 cones, is more concentrated in the ventral retina [25], but has distinct connectivity with postsynaptic interneurons [25–27]. Mice, therefore, have two types of UV-sensitive cones in direct proximity.

Similarly, some fish species including cichlids and salmonids are known to switch opsin expression in individual cones, such that PR1 cones may express LWS or RH2 opsins, and PR4 cones may express SWS1 or SWS2 opsins depending on developmental stage or environmental cues [28–30]. While these examples illustrate the problem, they are not outliers in the vertebrate tree of life. The identity and wavelength specificity of expressed cone opsins is subject to routine variation [4,19], both across species (e.g., [31]) as well as within species (including by retinal region [21,23], life stage [32], season [33–35], and environment [36]). It further depends on an opsin’s associated chromophore (A1 or A2) [37,38], which can also vary seasonably and according to life stage. In fact, opsins and their properties are evolutionarily labile traits, varying as species enter new visual niches [19,39]. The identity or functional properties of opsins therefore do not reliably specify the identity of the neuron that expresses them.

**Fig 2:**
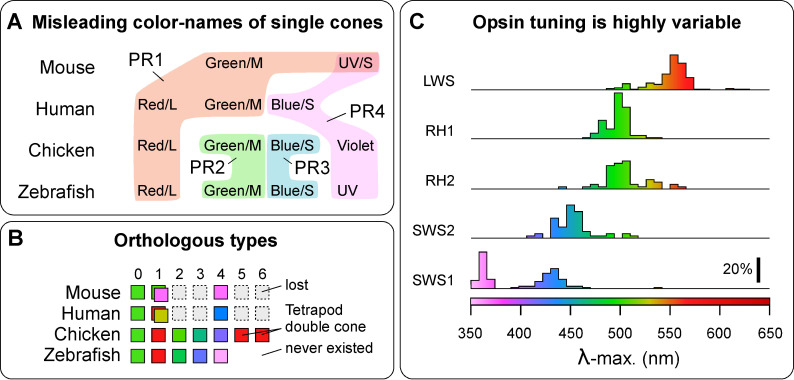
Issues with naming cones by color. **(A,B)** Cone ‘color names’ do not necessarily align with orthologous neuron types across vertebrates (**(B)** redrawn based on [9]). **(C)** Sequence changes within vertebrate opsins can give rise to diverse spectral properties (redrawn based on information in [19]). Note, for example, that a λ-max of approximately 500 nm is readily achieved by four out of five opsin families and the sensitivities of SWS1 opsins are bimodal, with one peak in the ultraviolet (i.e., <400 nm) and the other peak in the violet. Color shadings as defined on the bottom right by most commonly expressed opsin variant, independent of oil droplets, where present. Scale bar denotes percent of opsin variants as listed in [19]).

A second issue is that a definition by ‘color’ implies that wavelength selectivity is the only important characteristic of a photoreceptor. This is misleading [9], because beyond wavelength selectivity, cone types systematically differ in their basic cellular physiology, including their spatio-temporal properties [40–42], as well as in their developmental postsynaptic wiring [26,43,44] – all of which directly feed into their distinct roles in vision (Fig 2) [45].

### Cellular morphology is an imperfect indicator of photoreceptor identity

Morphological definitions of photoreceptor types based on the shape of the outer segment (i.e., ‘rod’ versus ‘cone’), association with other photoreceptors (e.g., ‘single’ versus ‘double’ cones) or other cellular features are equally problematic. Photoreceptors can be grouped into ‘morphological types’, namely ‘single cones’ which tend to occur in isolation, ‘twin’ cones [20] which comprise pairs made up of morphologically identical (or nearly identical) partners, and ‘double/triple cones’ consisting of asymmetric groups, often with ‘principal’ and ‘accessory’ members [11,20,46,47]. Single cones are occasionally further identified by other descriptors such as ‘long’, ‘short’ [48], and miniature [49] in reference to their size and/or vertical location in the outer retina. However, there are many factors that influence the anatomical arrangement of photoreceptors, and like opsin or spectral identity, none are reliably stable across or within species.

**Fig 3:**
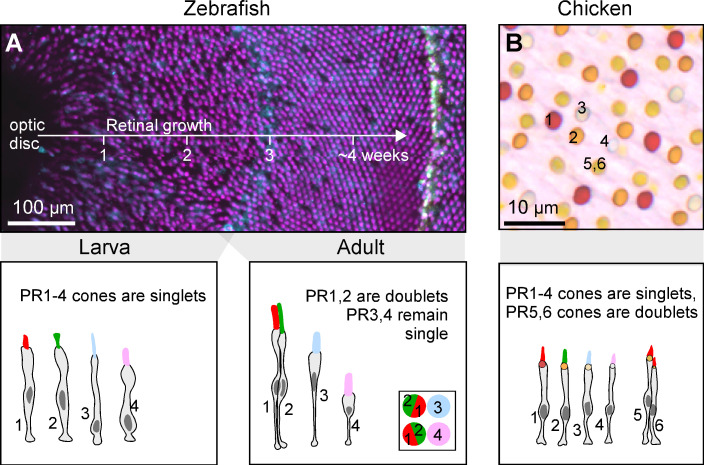
Zebrafish and chicken retinal structures in development. **(A)** Developmental transition of zebrafish cone patterning from a mosaic with low regularity to a highly regular row lattice (top, mod from [50]) and schematic summary of cone type arrangements at each stage (bottom, redrawn from [16,52,53]). **(B)** Oil droplets in chicken retina show five independent cone-type mosaics (top) and schematic summary of cone types (bottom, both mod. from [11]).

Zebrafish and chicken serve as two well studied examples (Fig 3). Adult zebrafish have a highly regular photoreceptor mosaic with PR1–4 cones arranged at a fixed 2:2:1:1 stoichiometry in the adult (Fig 1) [16,50]. These six neurons are arranged in a regular lattice whereby PR1,2 pairs alternate rows with PR3 and PR4. More generally, fishes exhibit diverse patterns of PR1–4 cones that vary in spectral content and regularity depending on the species, developmental stage and retinal location. PR1,2 pairs have been generally referred to as ‘paired cones’. If the two members have different morphology (usually with PR1 being larger than PR2) or visual pigment content, they have been called ‘double cones’. If they appear morphologically equal and contain the same visual pigment, they have been termed ‘twin cones’ [51].

In non-tetrapod vertebrates, all the above terms describe subsets of the same four ancestral cone types [22], PR1–4. One way to ascertain this is during development: In zebrafish, the retina does not start out in a near crystalline arrangement that forces some cone types into pairs. Instead, larval cones occur in independent mosaics [50,52,54]. This developmental history is inscribed into the adult retina as the ‘larval patch’ near the optic disc, which never rearranges [50,55]. Similar developmental patterns were also recently observed in several other fishes [56]. Beyond this evidence, the conclusion that all (zebra)fish cones are subsets of the same four ancestral ‘single’ cones is overwhelmingly supported by single-cell transcriptomics [22,57]. By contrast, many tetrapods, with the notable exception of eutherian mammals (and possibly snakes [58]), do have an ‘extra’ pair of cone photoreceptors, called ‘the tetrapod double cone’ [59], composed of the principal (PR5) and accessory (PR6) members. Unlike fish ‘double/twin/paired’ cones, the tetrapod double cones exist in parallel to PR1,2. Birds and most reptiles, therefore, have six ancestrally distinct types of cones: The four ‘original’ cones PR1–4 plus the two members of the tetrapod double cone PR5 and PR6. This view is supported by morphological [11,43,60,61], behavioral [47,62], functional [63,64], and molecular evidence [22,65,66].

## A time for change

To overcome such naming confusions, current progress in life sciences requires cell-type definitions that encompass a wide spectrum of features [67–69], ideally combining morphological [14], functional [70], developmental [22,71], and molecular traits [72,73]. We also emphasize the importance of deeply conserved transcription factors as type-defining features in our classification.

The vertebrate retina, with its planar structure and regularly tiled architecture has long served as a central model system for cell type taxonomy. Retinal neurons form spatially repeating units [74] that anchor efforts to define types and their plausible subdivisions across animal brains. In some species such as the mouse, the catalogue of retinal neuron types is probably close to complete. This momentous achievement has been gradually unlocked by the use of transgenic tools [12], large-scale physiological recording techniques [70,75], EM-connectomics [13,14,26], and, perhaps most importantly, single cell transcriptomics [68,69,72,76–78], which can assign a genome-wide molecular signature to anatomical and functional catalogues.

The bipolar cells of the mouse retina were amongst the first to be ‘solved’ in this way [26,76,79], and it has taken only a few more years for the tabulation of other retinal neuron types in a number of species to become increasingly complete. The mouse retina comprises approximately 130 neuron types: the rod, 2 cones, 1 horizontal cell, 15 bipolar cells [13,26,76,80], approximately 63 amacrine cells [77] and 45 ganglion cells [68–70,81,82]. Of these, all except the rods and cones are primarily known by a numbered identity (e.g., H1 horizontal cell, Type 1 bipolar cell, etc.). While aspects of these catalogues still require consensus and alignment across species, in principle a numerical system gives each type of neuron a unique and unmistakable identity. For example, a horizontal cell that is orthologous to the H1 of mice [83] also exists in lampreys [84], zebrafish [52,78], chicken [17,65,85], and humans [78,86]. This understanding allows us to make powerful inferences across the vertebrate tree of life.

Recent transcriptomic studies (e.g., [22,71,87,88]) investigated molecular relationships of vertebrate photoreceptors across a broad range of species. Among other findings, this work provided a molecular confirmation of what had long been suspected [9]: Vertebrate photoreceptor types appear to be orthologous across multiple evolutionarily distant species. We believe that the time has come to inscribe this knowledge into the names of photoreceptor types.

## Our proposal

As a clear and systematic nomenclature for vertebrate photoreceptors, we suggest a numbering scheme that simultaneously mirrors the cell’s evolutionary history, their typical relative abundance in the eye, their development, and their systematically distinct postsynaptic wiring patterns (Fig 1).

Ancestral red cones [9], renamed ‘Type 1 photoreceptors’, or ‘PR1′, are probably the least derived type of cone. In most non-avian vertebrate eyes, barring rods, PR1 are the most abundant, and probably also the most important type of photoreceptor [9,45,59]. PR1 typically, but not always (e.g., Ref [89]), expresses LWS-opsin, and the only sighted vertebrates thought to lack PR1 are rod-only species that typically live in extreme low-light conditions such as the deep sea. Human daylight vision is overwhelmingly driven by PR1 cones [90], which include both their ‘L’ and ‘M’ variants (PR1_L_, PR1_M_) [8]. In mice, PR1 includes both the green-sensitive dorsal (PR1_M_) and the predominantly UV-sensitive ventral (PR1_M/S_) cones [21,23,24]. In each case, the subscript can flexibly reference distinguishing features of a photoreceptor (e.g. PR1_LWS_, PR1_510 nm_, etc.).

Beyond PR1, the other three ancestral single cones [2,9] (ancestral green/RH2, blue/SWS2, UV/SWS1) are designated PR2–4, respectively. This order mirrors their typical spectral order [19], their transcriptomic relatedness to PR1 [22], and their anatomical wiring order in the retina (‘spectral block wiring’ [4,16]), as well as the order of their typical numerical abundance and relative sizes (PR1 ≥ PR2 ≥ PR3 ≥ PR4, e.g., in chicken [11] and adult zebrafish [91]). Moreover, this sequence mirrors the numerical order of cones’ postsynaptic targets, namely horizontal cells and bipolar cells. For example, the zebrafish H1 horizontal cell is the only one that connects with PR1 cones, alongside PR2,3 [52]. H2 then links with PR2–4 cones, while H3 links to PR3,4. Likewise, the Type 1 bipolar cell of mice is the only cone-bipolar cell that preferentially targets PR1 cones [26]. The numerical system also elucidates evolutionary loss of cone types, e.g., for eutherian mammals who retain only PR1 and PR4 [9].

After these original five photoreceptor types, the next most widespread photoreceptor type in vertebrates is the tetrapod double cone (except in birds where double cones are dominant) [47,59,61], and we suggest the designations PR5 and PR6 for the principal and accessory members, respectively. This system also mirrors their likely evolutionary order of appearance [59]. We posit that the double cone should receive two numbers (5 and 6) rather than one because: (i) it consists of two constituent members [22,61,65]; (ii) each member makes independent connections to postsynaptic targets [43]; (iii) the two members almost certainly differ in their physiological properties and roles in vision [47,60,63]; and (iv) while the two cell types may have evolved from distinct ancestral single cones [22,71], they are morphologically [43] and molecularly [22,71] distinct, and exist in parallel to PR1–4.

Beyond these established photoreceptor types, many amphibians have a second rod type known traditionally as the ‘green rod’ based on its microscopic appearance (and in contrast with the canonical or ‘red rod’) [92–94]. Tentatively designated PR7, the ancestry of this eighth type of photoreceptor remains unresolved. However, its exclusive presence in amphibians strongly suggests that unlike PR5/6, PR7 may have emerged after present-day amphibians diverged from other tetrapods. Beyond PR7, further hints of yet-to-be-defined photoreceptor types include a possible ‘extra’ type in some marsupials [95] and subsets of photoreceptors found in snakes [58,96] and geckos [97–99]. We posit that these, or others, should be added onto the end of the proposed scheme if and when appropriate.

Finally, we propose that rods be designated PR0. Despite rods’ substantial molecular differentiation from cones [22], both rods and cones share a common ancestry, and the presence of rods (PR0) and ancestral single cones (PR1–4) in cyclostomes [2,84,100] strongly suggests that both were present in the common ancestor of extant vertebrates. While some species feature additional rod-like photoreceptors, it remains unclear which, if any, represent genuine new types.

## Naming yet-to-be-identified photoreceptor types

We recognize that the list of photoreceptor types presented in Fig 1 is not exhaustive. For example, evidence suggests that the little skate, deep-sea fishes, marsupials, geckos, and snakes may possess unusual photoreceptor types that do not fall within any of the categories we propose. Furthermore, the photoreceptor types of only a minute fraction of the nearly 67,000 extant vertebrate species have been studied in any detail. Thus, the present classification system is intended to be open-ended, permitting the addition of more photoreceptor types as they are characterized.

The goal of our definition is to include ‘extra’ types that exist in parallel to the ancestral ones (such as tetrapod double cones PR5,6 or the amphibian ‘second rod’ PR7), but to exclude those that represent within-retina variation (e.g., human PR1_L_ versus PR1_M_).

**Fig 4:**
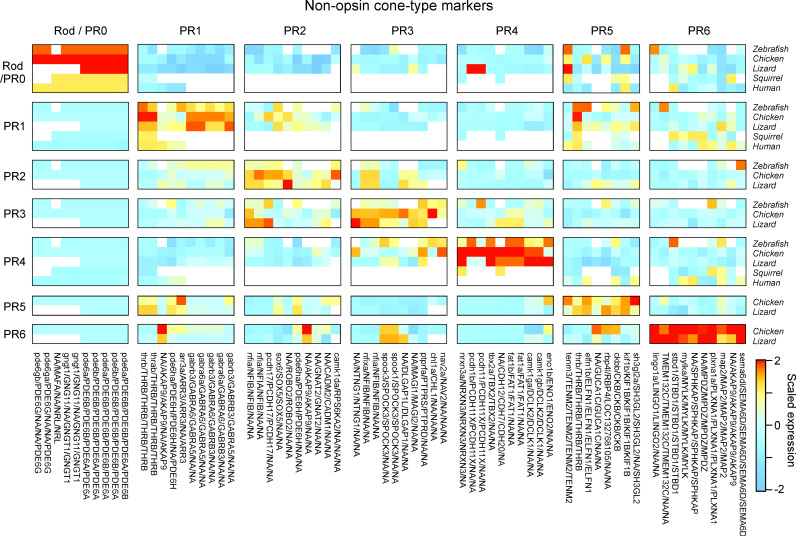
Non-exhaustive list of possible marker genes for rods and cones (redrawn based on [ 22**])**. Note that the second rod of amphibians PR7 is omitted because single-cell transcriptomic data remains outstanding.

More generally, the proposed definition of photoreceptors into ancestral types is centrally anchored in their distinct transcriptomic signatures [22] across vertebrate ‘model’ species that are thought to be broadly representative for their clade: Humans (primates), squirrel (rodents), chicken (birds), brown anole lizard (non-avian reptiles), and zebrafish (teleosts) (Fig 4). In these species, single-cell transcriptomic data conforms with long standing insights into a photoreceptor’s morphological and functional properties (reviewed in Refs [3,9,16,17,59]). Together, this wealth of data renders it unlikely that their cell-type definitions will need to be revised in the light of possible future evidence. We therefore posit that these species, and others where corresponding insights exist (e.g., Ref [84]), can serve as a reference when defining cone types in other species.

In addition, transcriptomic datasets are in good agreement with functional genetic experiments on the development of photoreceptor identity. These experiments have shown that specific transcription factors control fate decisions in photoreceptor progenitors and are thus required for the generation of each photoreceptor type or for the expression of their unique set of genes; transcriptomic datasets confirm that each photoreceptor type retains specific expression of these transcription factors throughout their life span, likely actively controlling cell-type identity. Furthermore, evidence suggests that transcription factors and their cognate binding sites throughout the genome are interlocked and therefore highly resistant to evolutionary change. Thus, transcription factors and *cis*-regulatory ‘grammars’ are often relatively fixed ancestral features of a cell type which are more persistent in evolution than the modules of ‘effector genes’ that they regulate, as the latter are more directly subject to present-day adaptive pressures [101,102]. Some examples of deeply conserved photoreceptor transcription factors are described in Box 1.

**Box 1. Deeply conserved photoreceptor transcription factors**

- Generation of rods/PR0 is dependent on Maf family transcription factors (e.g., NRL, MAFA, MAFB) and NR2E3 [103,104].
- Generation of PR1 depends on THRB, a subunit of the thyroid-hormone receptor [105–107],as well as the transcriptional co-factor SAMD7 [87].
- Generation of PR2 depends on SIX6/SIX7 [108,109].
- Generation of PR3 depends on FOXQ2; both PR3 and FOXQ2 were lost in eutherian mammals but retained in monotremes [110–113].
- Generation of PR4 has been shown to depend on TBX2 in zebrafish [88,114] and TBX2 expression is specific to PR4 in transcriptomic datasets across vertebrates (zebrafish, chicken, lizards, squirrels and primates).
- Generation of PR5 depends on THRB; and similar to PR1, PR5 also expresses SAMD7 [22,71].
- PR6 expresses FOXQ2 and SKOR1, similar to PR3 and PR3,4, respectively, and RXRG, similar to PR1 [22,71].

Aside from the above, inferences about photoreceptor identity can nevertheless emerge from limited data such as those used in classical definitions (Fig 1) and reference to typical patterns of photoreceptor properties across the vertebrate tree of life (reviewed, e.g., in [9,47,59,60]). For example, numerical abundance is usually PR1 ≥ PR2 > PR3 ≥ PR4. If present, PR5,6 is usually PR1 >PR5,6 > PR4, except in birds, where the more typical pattern is PR5,6 > PR1,2. If cone types are missing, the likely order of evolutionary loss is PR2 = PR3 > PR4 > PR1. In non-eutherian tetrapods, PR5,6 are usually present, and PR5,6 are not known to occur individually. In addition, postsynaptic wiring appears to conform to ‘spectral blocks’ in the sense that ‘intermediate’ cones, if present, do not tend to be skipped. For example, a bipolar cell is unlikely to connect with PR1 and PR3 without also contacting PR2. In this order, rods and PR5,6 appear to group with PR1 (i.e., PR0/PR5,6–PR1–PR2–PR3–PR4) [9,43]. In birds, PR6 appears to additionally group with PR3 [115]. Finally, the spectral appearance of pigmented oil droplets, if present, generally correlates with cone-type identity, with PR1–PR4 exhibiting long to short-wavelength filtering properties, respectively, correlating with the spectral sensitivity of the corresponding opsins. PR4 usually has a clear oil droplet, devoid of light-absorbing carotenoid pigments. PR5 tend to have spectrally intermediate oil droplets and PR6 tend to have either absent or minute droplets, while frequently retaining carotenoid pigmentation in the mitochondrial aggregates of the ellipsoid [60].

## A final word

We acknowledge that some common names for photoreceptor types are deeply engrained, both in the scientific literature but also in popular culture. For example, the terms of ‘L’, ‘M’, and ‘S’ cones of the human eye, although potentially misleading (discussed in Ref [9]), may persist in the foreseeable future. However, as a community we can endeavor to define the terms that we use in our communications by the simple and hopefully uncontroversial numbering scheme suggested here. Perhaps, in this way, it will eventually become more widely adopted.

## Abbreviations

**:**
PR: photoreceptor
UV: ultraviolet
LWS: long-wavelength sensitive
SWS: short-wavelength sensitive
